# Understanding the dynamics of crisis situations in care for community-dwelling older individuals—a qualitative study

**DOI:** 10.1093/ageing/afag288

**Published:** 2026-09-29

**Authors:** Dolly R E Haselager, Rosalinde K E Poortvliet, Miriam L Haaksma, Yvonne M Drewes, Oumaima Boulahfa, Hedwig M M Vos, Jacobijn Gussekloo

**Affiliations:** Department of Public Health and Primary Care, Leiden University Medical Center, Leiden, Netherlands; LUMC Center for Health and Ageing, Leiden University Medical Center, Leiden, Netherlands; Department of Public Health and Primary Care, Leiden University Medical Center, Leiden, Netherlands; LUMC Center for Health and Ageing, Leiden University Medical Center, Leiden, Netherlands; LUMC Center for Health and Ageing, Leiden University Medical Center, Leiden, Netherlands; Department of Internal Medicine, Division of Gerontology and Geriatrics, Leiden University Medical Center, Leiden, Netherlands; LUMC Center for Health and Ageing, Leiden University Medical Center, Leiden, Netherlands; Department of Internal Medicine, Division of Gerontology and Geriatrics, Leiden University Medical Center, Leiden, Netherlands; Department of Public Health and Primary Care, Leiden University Medical Center, Leiden, Netherlands; LUMC Center for Health and Ageing, Leiden University Medical Center, Leiden, Netherlands; Department of Public Health and Primary Care, Leiden University Medical Center, Leiden, Netherlands; Department of Public Health and Primary Care, Leiden University Medical Center, Leiden, Netherlands; LUMC Center for Health and Ageing, Leiden University Medical Center, Leiden, Netherlands; Department of Internal Medicine, Division of Gerontology and Geriatrics, Leiden University Medical Center, Leiden, Netherlands

**Keywords:** crisis situations, older adults, tipping points, ageing-in-place, care triads, qualitative research

## Abstract

**Background:**

Crisis situations in care for community-dwelling older individuals, where the capacity of existing care arrangements at home is suddenly and unsustainably exceeded, can lead to adverse outcomes, such as hospitalisation, institutionalisation and death. Understanding how these crisis situations arise and take shape may contribute to their prevention and mitigation.

**Objective:**

To investigate the dynamics through which crisis situations in care for community-dwelling older individuals develop and take shape, as reflected in the experiences of older individuals, informal caregivers and healthcare professionals.

**Methods:**

This qualitative study consisted of 45 semi-structured interviews about experiences with crisis situations among older individuals, informal caregivers and healthcare professionals from diverse disciplines, with two additional focus groups involving general practitioners. Data were analysed using thematic analysis.

**Results:**

The analysis yielded two main themes: ‘Tipping points’ and ‘Progressive instability in care systems’. The first theme captures how tipping points mark the moment when remaining at home becomes untenable because the situation is unsafe, undignified or because an acute event requires medical attention. The second theme shows that older individuals are part of care systems that can become progressively unstable through various processes.

**Conclusion:**

Crisis situations in care for community-dwelling older individuals are systemic phenomena, developing through progressive instability in care systems and taking shape at tipping points, when these systems suddenly lose their balance. This systemic understanding of crisis dynamics may help healthcare professionals map key issues, recognise early instability and identify resilience, thereby supporting crisis prevention and management.

## Key points

- Crisis situations are disruptive to the lives of older individuals and can lead to adverse outcomes.
- A crisis situation can be understood as a sudden imbalance in the care system an older individual is part of.
- Tipping points signify that a situation at home is unsafe, undignified or that an acute event requires medical attention.
- Strengthening care system resilience may help prevent and mitigate crisis situations.

## Introduction

Ageing in place, the ability to remain at home throughout later life, is often a priority for older individuals and a policy objective in many European countries [1, 2]. When older individuals are faced with health issues that pose challenges to independent living in the community, care provided by both informal caregivers and community health services can help to overcome these challenges. However, the burden that informal caregiving places on the well-being and resources of informal caregivers limits the amount of care they can sustainably provide [3]. The availability of community health services is constrained by pressures on public funding and a growing shortage of healthcare professionals [1]. Numerous barriers, such as eligibility criteria, waiting lists and personal contributions may hamper older individuals’ access to care that meets their needs [4]. Simultaneously, the number of older individuals living in the community, including those with multimorbidity and frailty, is rising in ageing populations worldwide, indicating that the demand for care at home is set to increase [5, 6].

In the context of these societal challenges, a particular issue that warrants attention is the occurrence of crisis situations in care for community-dwelling older individuals (hereafter referred to as crisis situations): situations in which the capacity of existing care arrangements at home is suddenly and unsustainably exceeded [7–12]. Crisis situations carry an increased risk of adverse health outcomes, including emergency department visits, hospitalisation, institutionalisation, functional decline and death [13–15]. They may occur when an older individual with frailty abruptly becomes more dependent after a stressor, when an important informal caregiver unexpectedly becomes unavailable, or when escalating behavioural symptoms in an individual with dementia become too challenging to manage at home [11, 12, 14, 16]. A crisis signifies a disruption in the life of an older individual and is often a stressful and difficult situation for informal caregivers and healthcare professionals too [11–13]. Organising, coordinating and delivering the immediate care needed during a crisis situation can be challenging due to the complexity of these situations.

Studies on crisis situations in care for community-dwelling older individuals have mostly focused on specific clinical contexts, particularly dementia care or on adverse outcomes for older individuals in acute care settings, such as unplanned hospital admissions [8–15]. Crisis as a broader phenomenon, including how such situations develop and take shape through interacting factors, has not been extensively studied. In this study, the processes and interactions through which a crisis develops and takes form are referred to as crisis dynamics. Understanding these dynamics is important for meeting older individuals’ care needs in these situations, for informing improvements in the organisation of care, and for addressing the challenges that these crisis situations entail for older individuals, informal caregivers and healthcare professionals. From a socio-ecological perspective on caregiving, these stakeholders collectively form a triadic care unit [17, 18]. This model goes beyond the binary distinction between those giving and receiving care. It recognises the distinct needs and responsibilities of each member of the unit and emphasises their interconnectedness through caregiving relationships, which are often reciprocal. In line with this model, a comprehensive understanding of crises requires consideration of the experiences and perspectives of the members of the triadic care unit. Therefore, the aim of this study is to investigate the dynamics through which crisis situations in care for community-dwelling older individuals develop and take shape, as reflected in the experiences of older individuals, informal caregivers and healthcare professionals.

## Methods

### Design

This qualitative study reports on focus groups with general practitioners and semi-structured interviews with all members of the triadic care unit: older individuals, informal caregivers and healthcare professionals from diverse specialties. The study was approached through a constructivist theoretical lens, viewing a crisis as an inherently personal experience of older individuals, informal caregivers and healthcare professionals, which is shaped by sociocultural context and language [19]. This study was designed and reported in accordance with the consolidated criteria for reporting qualitative research [20].

### Setting

The study was conducted in a densely populated urban area in the Netherlands. The Dutch healthcare system is characterised by universal health insurance, a strong primary care system with out-of-hours services, and publicly funded home-based care and long-term care services [21].

### Recruitment and data collection

For recruitment, a broad operational definition of a crisis situation was adopted: a care situation in which the capacity of existing care arrangements at home is suddenly and unsustainably exceeded, in any healthcare setting. Healthcare professionals from various disciplines were approached via e-mails, newsletters and printed promotional material distributed in regional care networks. They were invited to participate in an interview themselves and to refer the invitation to an older individual in their care who had experienced a crisis, and to that individual’s informal caregiver. Older individuals and informal caregivers were thus recruited through their healthcare professionals and were also approached through newsletters from the regional Older People Advisory Board Health and Well-being. Purposive sampling was applied, seeking variation in crisis setting, outcome and the issues described as central by participants, and for healthcare professionals also profession and work experience. Interviews were semi-structured using a topic list that prompted participants to provide detailed accounts of their experiences with crises and their perspectives on how these situations had developed, including how circumstances and contributing factors had changed and interacted over time (See Appendix 1 in the Supplementary Data section for the topic lists). The topic list was developed with expert input from community-based clinicians and was refined after being tested in a pilot interview. Interviews were conducted in Dutch, either face-to-face at the participant’s home or workplace, or through video communication services. Additionally, two focus groups with general practitioners were conducted, which provided complementary input through a range of broad case descriptions and, given the general practitioners’ continuous role in older individuals’ care, a connecting perspective that linked different parts of the care trajectory. Interviews were conducted by DH (female, PhD student and general practitioner trainee), RP (female, senior researcher and general practitioner) and OB (female, junior researcher), who all had previous interviewing experience and did not have a relationship to the participants prior to the interview. Interviews lasted on average 59 minutes (range 33–163 minutes). All interviews were audio-recorded, transcribed verbatim and accompanied by field notes made during or after the interview. Recruitment started in December 2023 and data collection was finalised in January 2025.

### Participants

The study included 45 interviews and two focus groups. Table 1 shows the types and number of interviews and focus groups conducted. Table 2 shows the characteristics of individuals who participated in the interviews and focus groups. The study sample comprised six older individuals who had experienced a crisis situation themselves, 16 informal caregivers who had experienced such a situation with the person they cared for, and 48 healthcare professionals who had professional experience with these situations. Among informal caregivers, their relationship to the person they cared for was that of a child, sibling, spouse or other relative. Among healthcare professionals, the professions represented included community-based nurse, emergency department nurse, dementia case manager, elderly care physician, general practitioner and hospital-based physician.

**Table 1:** Types and number of interviews and focus groups conducted.

Interview type	Number of interviews
Single older individual	4
Older individual + informal caregiver	2
Single informal caregiver	6
Two informal caregivers	4
Single healthcare professional	28
Two healthcare professionals	1
Focus groups with general practitioners	2

**Table 2:** Characteristics of individuals who participated in the interviews and focus groups.

		Number of individuals (%)
Older individuals		6 (100)
Sex		
	Female	3 (50)
	Male	3 (50)
Informal caregivers		16 (100)
Sex		
	Female	8 (50)
	Male	8 (50)
Relationship with the older individual		
	Child	6 (37.5)
	Other relative	2 (12.5)
	Sibling	2 (12.5)
	Spouse	6 (37.5)
Healthcare professionals		48 (100)
Sex		
	Female	12 (75)
	Male	36 (25)
Profession		
	Community-based nurse^a^	6 (12.5)
	Emergency department nurse	3 (6.3)
	Dementia case manager	5 (10.4)
	Elderly care physician^b^	5 (10.4)
	General practitioner	5 (10.4)
	General practitioner in focus group	17 (35.4)
	Hospital-based physician^a^	7 (14.5)

^a^Profession descriptions were generalised to ensure participant anonymity

^b^A medical specialty in the Netherlands focused on long-term care for older individuals with complex chronic conditions

### Data analysis

A thematic analysis was applied to the interview data, in which themes capturing patterns of shared meaning were constructed in an iterative process [19, 22]. ATLAS.ti software was used to aid the analysis. In the initial phase, DH and OB familiarised themselves with the data by listening to interview recordings, reading transcripts, making notes and mapping how crisis trajectories appeared to unfold. Subsequently, they inductively coded all transcripts, engaging in regular discussions to exchange ideas, compare interpretations of new codes, and consider how codes related to each other within and across interviews. Overlapping codes were merged, codes that were overly broad were subdivided, and related codes were grouped together to establish a code tree. Data saturation across interviews was reached when no new codes or insights emerged from further interviews. DH, OB, RP and HV (female, senior researcher, general practitioner) came together to review the codes and develop the preliminary themes, after which DH wrote a preliminary report outlining each theme. The research team subsequently discussed whether the preliminary themes were in line with the codes and reflected the overall meaning in the data. In the final phase, feedback from all authors was sought to refine the analysis, resulting in the final themes. Representative quotes were chosen to illustrate the themes.

### Reflexivity and ethics

Several members of the research team are physicians working in primary care and have encountered crisis situations in their own practice. We acknowledge that these experiences informed study design, in particular recruitment, and analysis. This background enabled us to closely relate to the experiences of healthcare professionals, positioning us to some extent as ‘insiders’. To strengthen the representation of older individuals and informal caregivers, we sought advice from the regional Older People Advisory Board Health and Wellbeing during the preparation and analysis phases, where they advised on the research protocol and topic list and reflected on the preliminary results. To enhance the rigour of the study, we kept reflective notes and held regular discussions with the research team. This study was approved by the research ethics committee of our institution (nWMO approval reference number 23-3119). All interview participants provided written informed consent. We used an AI tool (Copilot) to translate the Dutch quotations and topic lists (Appendix 1 in the Supplementary Data section) into English. All translations were manually reviewed and verified by the research team.

## Results

The analysis yielded two interrelated themes: ‘Tipping points’ and ‘ Progressive instability in care systems’, each comprising three subthemes. Table 3 provides an overview of the themes and subthemes. The first theme centres on the acute moment at which the care situation becomes untenable at home. The second theme then extends the analysis to the broader processes through which the balance at home can gradually become more fragile. Finally, the two themes are synthesised into a conceptual understanding of the dynamics of crisis situations, presented visually in Figure 1.

**Table 3:** Summary of themes and subthemes.

Theme	Subtheme	Summary
1. Tipping points		Tipping points are key moments through which crisis situations take shape and mark the onset or awareness of a crisis in time. They can be understood as concrete events and as moments that carry significance, indicating why a particular situation has become untenable at home.
	Acute events requiring medical attention	Tipping points can signify acute events that require medical attention.
	Unsafe	Tipping points can signify that a situation has become too unsafe to remain at home.
	Undignified	Tipping points can signify that a situation at home is no longer acceptable because it is undignified.
2. Progressive instability in care systems		Crises are systemic, arising from the overall configuration of needs, resources and care relationships within the triadic care unit, which together form a care system. Crises develop through processes of progressive instability of these care systems, ultimately leading to a loss of balance and the onset of crisis.
	Cascade of health-related problems	In the period before a crisis, a cascade of health-related problems can place increasing strain on the care system.
	Presence and resilience of informal caregivers	The presence and resilience of informal caregivers shape the care system’s capacity to absorb emerging problems.
	Access to and mobilisation of professional care	Difficulties in accessing and mobilising professional care contribute to the instability of the care system in the period leading up to a crisis.

**Figure 1:**
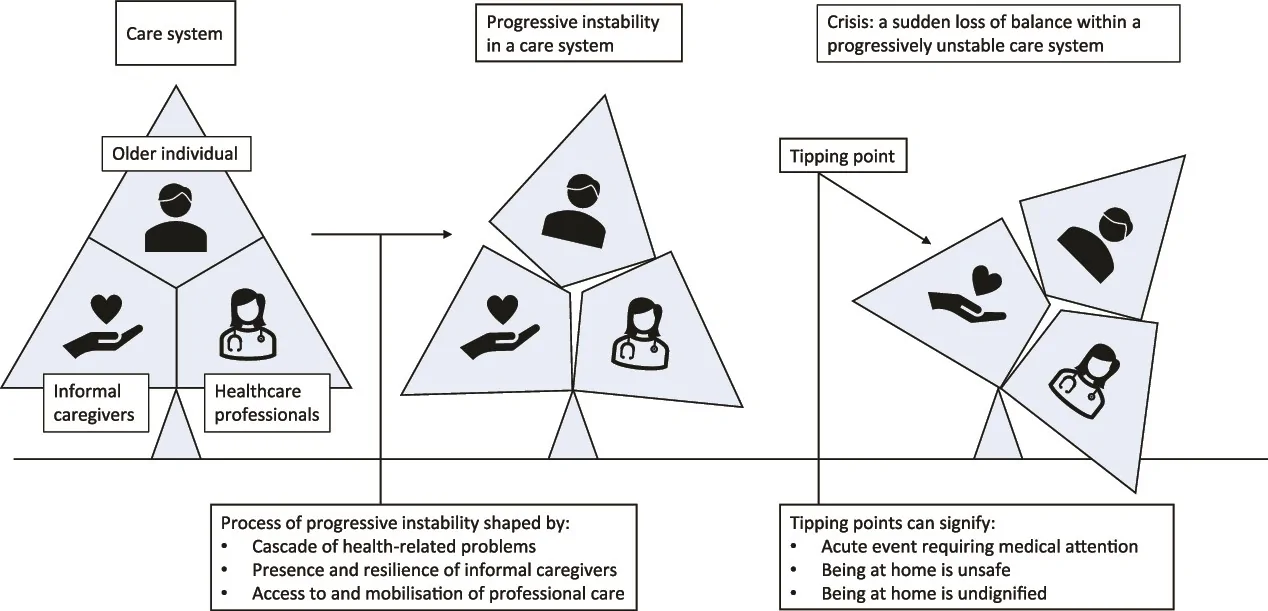
Conceptual understanding of the dynamics of crisis situations in care for community-dwelling older individuals. This figure illustrates how a crisis emerges from progressive instability within a care system formed by the triadic care unit, eventually leading to a loss of balance. The tipping point, which may arise in any component of the care system, signals the onset or awareness of this loss of balance.

### Theme 1. Tipping points

Participants described moments that marked the beginning of the crisis situations they had experienced. These moments signalled the point at which the capacity of existing care arrangements was suddenly and unsustainably exceeded, a point that can be understood as the tipping point. The experiences of participants indicated that tipping points are key moments within the dynamics through which crisis situations take shape, and are therefore a defining feature of the crisis itself. These tipping points can be understood both as concrete events and as moments in which any member of the triadic care unit recognises that the situation had become a crisis. Participants often summarised the tipping point with the Dutch phrase *‘Zo gaat het niet thuis’*, an expression used to convey that the situation at home had become untenable and urgent.

Emergency department nurse (interview 3):

‘She had fallen several times at home and was covered in bruises. […] What it really came down to was that the situation at home had become untenable. The family kept stressing this too.’

The accounts of participants showed that tipping points as concrete events often involved new health issues, exacerbations of chronic conditions or progression of diseases, leading to a sudden increase of care needs. Such events acted as destabilising moments that disrupted the existing balance at home.

Elderly care physician (interview 11):

‘If there is a urinary tract infection or influenza, or whatever, then it collapses like a house of cards.’

In some crisis situations, the tipping point was a major event, whereas in others a subtle change in an older individual’s health status became the tipping point, a small but decisive shift that marked the onset of the crisis.

Hospital-based physician (interview 30):

‘What I often notice is that nothing major has really changed recently, but that it’s more the final straw that makes the situation no longer manageable at home.’

Tipping points could also involve a sudden change in the availability of care and support, for example when a key informal caregiver became unexpectedly unavailable. In these situations, the care needs of the older individual did not change, but a crisis arose because these needs could suddenly no longer be met at home.

Community nurse (interview 10):

‘We had an older couple in our care. The husband had dementia, and it was just about manageable. […] But in the end, it was his wife who went first. […] Yes, she passed away, and he was admitted in a crisis situation, because he absolutely couldn’t live at home on his own.’

The experiences of participants also showed that tipping points did not always involve a clearly defined event. In some cases, they were the moment in which any member of the triadic care unit recognised that an ongoing situation had become a crisis, highlighting the subjective character of such moments.

Elderly care physician (interview 27):

‘I also find it difficult, because it can be quite subjective at times. And it really depends on who considers it a crisis.’

Beyond identifying tipping points as moments, participants also described the significance these moments held, essentially answering *why* a particular care situation had become untenable at home. This significance took different forms, including acute events requiring medical attention, a sense that a situation had become unsafe, or a sense that the situation had become undignified.

#### Subtheme: Acute events requiring medical attention

Participants’ accounts showed that tipping points could signify acute events that required medical attention and left the older individual too ill to remain at home. Participants also described situations in which these acute events were not immediately apparent. Some older individuals and informal caregivers recounted presenting to urgent care services because they could no longer manage care at home, and only after assessment did it become clear that an underlying acute event necessitated hospital-level care.

Older individual (interview 8):

‘I told my sister, please call them [the home care team]. And that happened. I was just lying there, and then the general practitioner came. And it turned out it was bad. I had pneumonia and on top of that, influenza, they found out in hospital.’

Healthcare professionals similarly noted that when older individuals presented to acute care services in crisis with non-specific complaints, the tipping point could lie in an acute event requiring medical attention. For this reason, they emphasised the importance of conducting a thorough somatic evaluation when older individuals presented to urgent care services in crisis.

Hospital-based physician (interview 24):

‘There’s this category of patients, something happened, fell down the stairs and now the situation at home is untenable. That, of course, very rightly belongs here [at the emergency department], to at least examine that part: falling down the stairs, or a urinary tract infection, or fever, legs giving way, for a somatic assessment.’

#### Subtheme: Unsafe

Participants’ experiences indicated that tipping points could also signify that the home environment had become unsafe, meaning that the risk of harm or negative health consequences had become too high to remain at home. Such tipping points included situations with a high risk of falls, an inability to call for help during an emergency, or potential negative health consequences arising from difficulties managing nutrition, hydration or personal hygiene.

General practitioner (interview 9):

‘The issue was that he would stand up and start doing things, and then he’d fall, he kept falling. He hadn’t broken anything or the like, so there was none of that. But he was falling, likely due to, well, hallucinations, I think. And he was more confused than before. […] And, of course, that’s clearly unsafe. […] So yes, suddenly, on a Sunday morning, the problem had to be resolved.’

Tipping points related to unsafety also arose from behavioural symptoms, particularly among older individuals with dementia or delirium. Some of these symptoms created danger for the older individual themselves, such as wandering, while others posed risks to people around them, including verbal or physical aggressive behaviour. In some situations, the level of danger created by these behavioural symptoms led to a compulsory crisis admission when the older individual lacked decision-making capacity.

Spouse (interview 41):

‘It actually escalated one evening, […]. Then she snapped and came at me. There was a glass table there. I pushed her away, I can’t get up that quickly, and she fell onto that table. I have an alarm button, so I immediately pressed it.’

#### Subtheme: Undignified

Participants’ accounts indicated that tipping points could also signify that a situation at home had become undignified. Participants described moments in which older individuals were no longer able to perform basic personal tasks and lacked available support to assist them, leading to situations they perceived as no longer acceptable at home. These situations involved older individuals who could no longer feed themselves, required continuous guidance because of cognitive impairments, or experienced incontinence and were unable to clean themselves adequately.

Child (interview 36):

‘But I find that inhumane. […] To, so to speak, leave someone sitting in their own filth… I’m sorry, but that’s just something you don’t do to anyone.’

For participants, these situations did not only reflect a loss of dignity for the older individual; they also marked a personal boundary of what they themselves felt was acceptable.

Hospital-based physician (interview 20):

‘We recently had someone with advanced dementia who had fallen on the street and was basically found and picked up, and they had no family, nothing. […] Of course, you can’t send someone like that home. […] I think that’s really inhumane. For me personally, and I think we all feel this way, but of course, I can’t be sure, that’s where we draw a line.’

Normative terms such as inhumane, undignified or unsafe were mainly used by healthcare professionals and informal caregivers. Older individuals described comparable situations through more concrete accounts that included feelings of vulnerability, disorientation or shame.

Older individual (interview 34):

‘Apparently I had been lying in the little hallway next to the bathroom. My son and one of my sisters […] found me there, with my bare bottom exposed. […] Even if I had had ten alarm buttons, no one would have come to help me. […] I feel ashamed that something like that happened.’

#### Theme 2. Progressive instability in care systems

Participants’ accounts showed that crisis situations arose in highly diverse circumstances, indicating that their development cannot be understood by examining contributing factors in isolation from the context in which they occurred. For example, crises occurred in care situations with little or no informal support but also when many informal caregivers were involved, and in older individuals with substantial care needs but also in those who had been largely independent until the crisis arose. Instead, crises appeared to be systemic, emerging from the overall configuration of needs, resources and care relationships within the triadic care unit. This configuration can be understood as a care system, in which the care needs of an older individual are addressed through the combined efforts of the older individual, informal caregivers and healthcare professionals. The experiences of participants showed that the development of crisis situations was driven by progressive instability in these care systems. These dynamics could ultimately lead to a loss of balance and the onset of a crisis. Processes of progressive systemic instability were shaped by a cascade of health-related events, by the presence and resilience of informal caregivers, and by the extent of access to and mobilisation of professional care.

#### Subtheme: Cascade of health-related problems

Participants’ accounts suggested that, before the crisis arose, a cascade of health-related problems placed increasing strain on the care system and undermined its stability. Older individuals and informal caregivers described a gradual accumulation of health-related problems in the period before the crisis, ranging from episodic events such as falls or hospital admissions to a progressive decline in the ability to perform daily tasks at home.

Relative (interview 37):

‘Things had obviously not been going well at home for months. […] How often the home care staff had to pick her up, we even have photos of it, she had fallen again during the night and then just sat there the whole time, until someone came to help her up.’

Healthcare professionals noted that health-related problems contributing to crisis situations had often been present for an extended period. They described how the accumulation of health-related problems over time left older individuals living at home in a fragile balance, with everyone involved trying to adapt to increasing needs. This cascade stretched the limits of the care system’s adaptability and appeared to ultimately compromise its stability.

Hospital-based physician (interview 15):

‘I am thinking about patients whom we see fairly regularly in the emergency department. People who are already vulnerable, who are still living independently at home, but for whom there is already a fragile balance, where it doesn’t take much disruption in their health, or otherwise in their situation, for them to no longer be able to manage at home.’

#### Subtheme: Presence and resilience of informal caregivers

The experiences shared by interview participants indicated that the presence and resilience of informal caregivers shape the care system’s capacity to absorb emerging problems. Healthcare professionals observed that when informal caregivers were consistently involved, they often buffered arising issues and prevented escalation into crisis. In contrast, older individuals with limited informal support were vulnerable to experiencing a crisis, as they had no caregivers to notice emerging problems, assist with daily activities, advocate for their wellbeing, provide a sense of safety, or respond promptly to emergencies.

Emergency department nurse (interview 3):

‘Then you have an 86-year-old mother who comes in, followed by six or seven children, all of whom know exactly how she is doing and who are all juggling things and keeping everything going. They really do come up with very adequate solutions. […] In other words, the system does not collapse all at once.’

Participants noticed that informal caregiving tasks were not exclusively performed by direct family members. Neighbours and community members often offered support and kept an eye on one another. These accounts showed that informal caregiving took shape as a wider network of relationships, with this broader network supporting the care system’s overall balance.

Elderly care physician (interview 35):

‘People who have a network are able to manage living at home for longer. You see this with informal caregivers or children who live nearby, sometimes even in the same household, and in senior housing settings, with more social oversight. If someone is lying on the floor, it is noticed.’

Participants also described how the demands of caregiving could become increasingly difficult to sustain, with informal caregivers’ resilience stretched beyond its limits. Informal caregivers recalled how they were hesitant to seek professional assistance when caregiving became increasingly overwhelming, motivated by the long-standing and close relationships they had with the individuals they cared for.

Spouse (interview 45):

‘I really thought, it is getting quite intense. I promised that I would stay with him, right, that I would take care of him. […] We had two ladies who helped here, but those ladies… well, my husband didn’t want that at all. He wanted me.’

These accounts showed that the care system’s reliance on the presence and resilience of informal caregivers is both a source of stability and a point of vulnerability. When informal caregivers become overwhelmed, the balance that had been maintained can quickly give way.

#### Subtheme: Access to and mobilisation of professional care

The experiences of participants showed that access to and mobilisation of professional care could, when available, help to stabilise the care system in the period before a crisis. Participants described how additional support, such as home adaptations and extra home care, often helped to keep things going at home. Conversely, difficulties in accessing and mobilising professional care could contribute to progressive instability in the care system. These difficulties meant that some older individuals were ‘flying off the radar’*,* as they were not known to, or recently assessed by, healthcare professionals, which participants viewed as a missed opportunity for early intervention and as a factor that made crisis situations more difficult to resolve. In contrast, when older individuals were already in view, healthcare professionals found it easier to respond to emerging problems, as they already had a clear understanding of the older individual’s situation, needs and preferences.

Hospital-based physician (interview 15):

‘It would help if we had people in view a bit earlier, so that when things do fall apart, as they sometimes do, we at least know what the situation is.’

Participants’ accounts showed that difficulties in accessing and mobilising professional care arose from a range of circumstances. Older individuals and informal caregivers reported that when care needs increased, they sometimes struggled to identify available care services and understand how to access them. For some, personal financial contributions posed a barrier to obtaining additional care. Healthcare professionals similarly observed that the complexity of the healthcare system, with its multiple entry points, impeded access, particularly for older individuals and informal caregivers with limited health literacy. They also noted that arranging care was especially difficult for older individuals who did not fit standard care categories, and that access could be constrained by scarcity of resources, reflected in waiting lists and eligibility criteria for home care and nursing home placements. Healthcare professionals also described how fragmented information across organisations and providers hindered communication and collaboration, especially in complex cases where responsibility for coordination was unclear.

Elderly care physician (interview 11):

‘It’s quite complicated. Well, you’d want a single point of contact where you could arrange everything, but you have to buy a walker yourself, and a shower chair or wheelchair you can only rent temporarily. So, how are you supposed to manage all of that as a patient, so many different systems? I find that quite difficult myself.’

The experiences of interview participants showed that older individuals and informal caregivers may also have personal reasons for not seeking additional professional care. Older individuals who experienced a crisis themselves described that they highly valued their personal autonomy and wished to remain in their own homes for as long as possible. Some shared that they were hesitant to seek help because they wanted to maintain their independence or because they did not want to burden others.

Older individual and child (interview 39):

Child: ‘It’s also about having control over yourself and what you do. You always want to keep going and maintain your own autonomy. That’s really the main issue here.’

Older individual: ‘Yes, I believe that’s what I’m known for.’

Healthcare professionals also noted a distinct group of older individuals who actively avoided care or declined available support, leaving professionals unable to assist until a crisis occurred. Finally, some participants mentioned that a crisis could also serve as a starting point for change, meaning that access to appropriate professional care only became possible once the crisis had emerged.

### Conceptual model

Taken together, the themes and subthemes can be synthesised in a conceptual understanding of the dynamics of crisis situations (Figure 1). A crisis can be understood as a sudden loss of balance within the progressively unstable care system an older individual is part of, with a tipping point, which can occur in any component of the care system, marking the onset or awareness of this imbalance.

## Discussion

The aim of this study was to investigate the dynamics through which crisis situations in care for community-dwelling older individuals develop and take shape, as reflected in the experiences of older individuals, informal caregivers and healthcare professionals. In the first theme, we showed that tipping points are key moments within these dynamics, marking the onset or awareness of a crisis in time and signifying either an acute event requiring medical attention or a situation at home becoming unsafe or undignified. In the second theme, we showed that crises appeared to be systemic phenomena, arising from the overall configuration of needs, resources and care relationships, which together form a care system. The dynamics through which crisis situations develop involve processes of progressive instability of these care systems, ultimately leading to a loss of balance and the onset of a crisis.

The idea of a crisis as a loss of balance has previously been proposed in research focused on older individuals with dementia [11]. Our study extends this perspective to consider the system in which this imbalance occurs. This systemic conceptualisation adds to a growing body of research in which a socio-ecological approach is used to study complex caregiving dynamics [23–26]. The dynamic of progressive instability in a care system resonates with studies using complex systems approaches to crisis situations and functional ageing [27, 28]. Our observations on tipping points are consistent with previous studies on crisis situations among older individuals, which describe both diverse health problems and carer absence as potential factors causing the onset of a crisis [7–12]. Our study adds that these tipping points carry meaning that is often centred on the concepts of safety and dignity. In research on compulsory crisis admissions in psychiatry and dementia care, these concepts, especially safety, are well established as central to a crisis [29, 30]. Our findings suggest that crossing a threshold in terms of safety and dignity may be relevant across a wide range of crisis situations in care for community-dwelling older individuals. Recognising safety and dignity as central to a crisis may help healthcare professionals unpack statements such as ‘we cannot manage at home,’ acknowledge the underlying concerns explicitly, thereby creating space to identify shared priorities with the older individual and informal caregivers involved and explore potential ways forward. At the same time, it raises questions about how safety and dignity are interpreted and weighed alongside other values, particularly autonomy, which underpins older individuals’ motivation to remain at home for as long as possible. It also raises questions about the extent to which healthcare professionals can, or should, be responsible for ensuring an older individual’s safety and dignity at home.

Understanding crises in the care for community-dwelling older individuals as a systemic loss of balance may offer new directions for practice. For healthcare professionals, this systemic lens may support the mapping of complex care situations, enabling them to see the broader picture. Understanding crisis situations as systemic may help them identify emerging instability within the care system and recognise where resilience within the system may contribute to restoring balance. Resilience, understood as the capacity of a person or system to respond to stressors while maintaining or restoring balance, may also be proactively strengthened in care systems where healthcare professionals signal instability [28]. The conceptual model of a progressively unstable system, as shown in Figure 1, may serve as a conversational tool, bringing older individuals, informal caregivers and healthcare professionals together to better understand the vulnerabilities and resilience of the care system they are part of, providing a starting point for proactive care. At the same time, even robust care systems can lose their balance when confronted with a sufficient stressor, such as an acute medical event. This underscores the need for urgent care services to be organised in ways that are attuned to the needs of older individuals who present in a crisis.

Strengths of this study include the integration of experiences of older individuals, informal caregivers and healthcare professionals, illuminating the dynamics of the triadic care unit, and the large number of interviews reflecting diverse real-world experiences. Another strength of this study is the exploratory approach, which enabled us to capture the heterogeneity of crisis situations and to investigate the dynamics of an idiosyncratic phenomenon that is widely recognised in practice but has not been extensively studied. As such, our findings offer a conceptual foundation for future research aimed at further unpacking the complex mechanisms shaping crisis situations in care of community-dwelling older individuals. Future studies might adopt a longitudinal approach to gain further insight in the dynamics of crisis situations. A limitation of this study is the relatively small number of older individuals included, which may have influenced the prominence of their perspectives in the findings. In light of this limitation and the study’s focus on crisis dynamics, the findings should be understood as developed through the integration of experiences and perspectives across the care triad, rather than as providing an in-depth account of a single participant group’s experiences. Another limitation is that, given the exploratory aim of our study, we did not collect data on socioeconomic position or cultural background of interview participants, though we acknowledge that structural inequalities and culturally shaped values and perceptions of care dynamics may influence both the development and the experience of crises, which warrants further examination in future research.

We conclude that crisis situations in care for community-dwelling older individuals develop through processes of progressive instability of the care systems they are part of and take shape through tipping points that mark the onset of a sudden imbalance within these systems. This systemic perspective on the dynamics of crisis situations in care for community-dwelling older individuals may support healthcare professionals navigate these complex care situations, helping them to map key issues, recognise early instability, identify points of resilience and facilitate shared understanding among those involved in the care system. Future research and policy aimed at strengthening the overall resilience of older individuals’ care systems might support both the mitigation and management of crises. In light of ageing populations worldwide, it is essential to address the dynamics of crisis situations in care for community-dwelling older individuals and develop care and policy strategies that truly meet these older individuals’ needs.

## Supplementary Material

aa_26_1575_File002_afag288
