# Supplementary material for: Understanding the dynamics of crisis situations in care for community-dwelling older individuals—a qualitative study
Source: Age Ageing. 2026 Sep 29;55(9):afag288. doi: 10.1093/ageing/afag288 (PMC13619340; doi:10.1093/ageing/afag288)
Supplement: Supplementary file 1 [file aa_26_1575_file002_afag288.docx]

**Supplementary Data File for: Understanding the dynamics of crisis situations in care for community-dwelling older individuals - a qualitative study**

**Appendix 1. Topic Lists**

**Contents**

1. Topic list for interviews with older individuals and informal caregivers
2. Topic list for healthcare professionals
3. Topic list for the focus groups with general practitioners
1. **Topic list older individuals and informal caregivers**

Introduction

- Introductions (interviewer introduced themselves with name and occupation)
- Explanation of the study
- Interview procedure and audio‑recording
- Opportunity for questions
- Completion of consent form

Topics (in no particular order)

- General health of the older individual
  - Physical well‑being
  - Mental well‑being
  - Social environment and network
  - Daily activities
  - Help and care
  - Living situation
- Changes in health
- Crisis situation
  - Period leading up to the crisis
  - Cause
  - Setting and people involved
  - Problems present at the time
  - Course of events
  - Outcome
- Help sought during the crisis situation
  - Role of healthcare professionals
- Reflections and experiences
  - Emotions or concerns
- Suggestions for improvement
- Other topics raised by participants

Closing

- Thanking the participant
- Ending the audio‑recording

2. **Topic list for interviews with healthcare professionals**

Introduction

- Introductions (interviewer introduced themselves with name and occupation)
- Explanation of the study
- Interview procedure and audio‑recording
- Opportunity for questions
- Completion of consent form

Topics (in no particular order)

- Crisis situations
  - Presenting complaint
  - How the professional became involved
  - Period leading up to the crisis (if known)
  - Contributing factors to the crisis situation
  - Setting
  - Timing
  - Problems present at the time
  - Course of events
  - Outcome
- Involved professionals and collaboration
- Professionals involved in the case
- Role of the participant
- Collaboration between professionals
- Reflection
- Professional’s reflections on the case
  - Emotions or concerns
  - Key learning points from the case
  - Suggestions for improvement
  - Perceived opportunities for improvement or change in care
- Other topics raised by participants
- General reflections on crisis situations
  - Frequency of similar cases
  - Common factors across cases
  - Perceived risk factors
  - Possibilities for earlier recognition
  - Possibilities for prevention
- Training and education
  - Training needs related to crisis situations

Closing

- Thanking the participant
- Ending the audio‑recording

3. **Topic list for focus groups with general practitioners**

Introduction

- Round of introductions (interviewer introduced themselves with name and occupation)
- Explanation of the study
- Focus group procedure and audio‑recording
- Opportunity for questions
- Completion of consent form

Topics (in no particular order)

- First thoughts about crisis situations in care for community-dwelling older individuals
- Examples from practice
- Typical cases encountered in practice
- Reflections on the examples
  - Core problems
  - Tipping points
- Role of family and other informal caregivers
- Experiences with cooperation with other healthcare professionals
- Challenges in managing crisis situations
- Other topics raised by participants

Closing

- Thanking the participants
- Ending the audio‑recording
